# Patient specific instrumentation in ACL reconstruction: a proof-of-concept cadaver experiment assessing drilling accuracy when using 3D printed guides

**DOI:** 10.1007/s00402-023-05072-w

**Published:** 2023-09-29

**Authors:** Mark J. M. Zee, Peter A. J. Pijpker, Joep Kraeima, Alain R. Viddeleer, Ronald L. Diercks

**Affiliations:** 1 Department of Orthopedic Surgery, University of Groningen, University Medical Centre Groningen, Hanzeplein 1, PO Box 30.001, 9700RB Groningen, The Netherlands; 2 3D Lab, Department of Orthopedic Surgery, University of Groningen, University Medical Centre Groningen, Hanzeplein 1, PO Box 30.001, 9700RB Groningen, The Netherlands; 3 3D Lab, Department of Oral and Maxillofacial Surgery, University of Groningen, University Medical Centre Groningen, Hanzeplein 1, PO Box 30.001, 9700RB Groningen, The Netherlands; 4 Medical Imaging Center, Department of Radiology, University of Groningen, University Medical Centre Groningen, Hanzeplein 1, PO Box 30.001, 9700RB Groningen, The Netherlands

**Keywords:** Patient specific instrument, ACL reconstruction, Anatomic, Femoral tunnel

## Abstract

**Introduction:**

Accurate positioning of the femoral tunnel in ACL reconstruction is of the utmost importance to reduce the risk of graft failure. Limited visibility during arthroscopy and a wide anatomical variance attribute to femoral tunnel malposition using conventional surgical techniques. The purpose of this study was to determine whether a patient specific 3D printed surgical guide allows for in vitro femoral tunnel positioning within 2 mm of the planned tunnel position.

**Materials and Methods:**

A patient specific guide for femoral tunnel positioning in ACL reconstruction was created for four human cadaveric knee specimens based on routine clinical MRI data. Fitting properties were judged by two orthopedic surgeons. MRI scanning was performed both pre- and post-procedure. The planned tunnel endpoint was compared to the actual drilled femoral tunnel.

**Results:**

This patient specific 3D printed guide showed a mean deviation of 5.0 mm from the center of the planned femoral ACL origin.

**Conclusion:**

In search to improve accuracy and consistency of femoral tunnel positioning in ACL reconstruction, the use of a patient specific 3D printed surgical guide is a viable option to explore further. The results are comparable to those of conventional techniques; however, further design improvements are necessary to improve accuracy and enhance reproducibility.

## Introduction

In young active patients who have suffered a rupture of the anterior cruciate ligament (ACL), ACL reconstruction is used to treat symptomatic knee instability [17]. Anatomical ACL reconstruction aims for a graft to be implanted on the native footprints of the ACL on the femur and tibia. Non-anatomical placement of the graft in ACL may eliminate anterior/posterior laxity, but normal kinematics will not be fully restored [3, 14, 23]. Also, non-anatomic placement of the ACL graft is associated with an increased risk of graft failure [11]. This graft failure, rupture, or elongation, occurs in up to 14% of primary ACL reconstructions [11] and does not depend on the type of graft used [9]. To reduce graft failure, it is important to address additional posterolateral, posteromedial and collateral laxity [26], but in up to 24% of patients that undergo ACL revision surgery, surgical inaccuracy is the sole reason for failure. And in up to 54% of patients, this is an additive cause for failure [7]. Malposition of the femoral tunnel is recognized as the most common technical failure (80%) [7]. Possible contributing factors are procedure and patient dependent: During the procedure, limited visibility of the femoral footprint during arthroscopy is a known problem [1, 25] and studies show that there is a large individual variation in location and diameter of the femoral footprint of the native ACL [28]. Although femoral and tibial bone tunnels are drilled through surgical guide instruments to optimize positioning, current surgical techniques still depend on the intra-operative identification of landmarks and measurements to determine the femoral footprint of the ACL. The use of anatomical landmarks for ensuring anatomic positioning of the graft however remains associated with a high risk of femoral tunnel malposition, which is related to early to midterm failure of the graft [7, 11]. This emphasizes that current surgical techniques using universal aiming devices seem to fall short in creating a constant and reliable result for a femoral tunnel position at the optimal, individual anatomic footprint of the ACL. To provide consistent results, determining the location of the ACL footprint should not be dependent of surgeon’s experience or intra-operative visual control, and individual variation should be taken into account.

To individualize anatomical femoral tunnel placement and thus improve graft survival, we developed a novel surgical aiming device to create a femoral tunnel at the individualized anatomic ACL footprint during ACL reconstruction. The use of this patient specific instrumentation in ACL surgery aims for a constant and reliable method to assure a femoral tunnel emerging at the native ACL position. Moreover, patient specific instrumentation can be of aid in complex revision cases with multiple previous bone tunnels and in cases with posttraumatic or torsional deformities of the distal femur.

In this cadaveric study, the in vitro accuracy of a patient specific 3D printed surgical guide, to be used for femoral tunnel positioning in an outside-in ACL reconstruction, was determined. The aim of this study was to drill a femoral tunnel in the specimen, emerging within 2 mm of the femoral footprint of the ACL, as determined by planning on preoperative MRI.

## Materials and methods

In this experiment, four knee joints of fresh frozen human cadavers were used. The study protocol has been reviewed by the Review Board of the University Medical Center Groningen (UMCG, Groningen, the Netherlands, study number 2015/057), and the committee has confirmed that no ethical approval was required. The cadavers were obtained from the Anatomy department of the UMCG. Knees with previous surgical procedures were excluded. Specimens were separated approximately 30 cm above and below the joint line. After 48 h of defrosting, the knees were scanned using an MRI scanner.

### Image acquisition

The specimens were placed supine in a patella forward position and fixed in a common knee coil. A 1.5 Tesla MAGNETOM® Aera MRI scanner (Siemens Healthcare GmbH, Erlangen, Germany) was used to acquire all scans. The used scanning protocol consisted of a routine clinical 2D knee sequence. The protocol consisted of Proton Density (PD) series in the sagittal, coronal, and axial planes. The use of PD series was chosen because of the more pronounced difference between the cartilage and the surrounding structures on these images. Voxel size of 0.4 × 0.4 × 3.0 mm was selected with a field of view of 160 mm, a flip angle of 150˚, a repetition time of 3530 ms., and an echo time of 41 ms. The scanning protocol used in this study was similar to the routine clinical protocol for diagnosing ACL injury. This avoids the need for additional scans when this concept is used for clinical purposes in the future. Files were saved for further processing in 16-bit Digital Imaging and Communications in Medicine (DICOM) file formats.

### Segmentation procedure

Using the Mimics Innovation Suite Software version 21.0 (Materialise, Leuven, Belgium), the images were segmented to obtain accurate 3D models of the knee. The MRI images were semi-automatically segmented with the use of the *livewire* technique as previously described [29]. Using this technique, the software is able to semi-automatically distinguish different gray scales in order to select a region of interest. The region of interest consisted of the femur including the overlying cartilage. Intra-observer reliability for the segmentation method was evaluated using repeated segmentations. The total absolute mean distance between models was 0.20 mm. Although the correctness of the 3D model was not evaluated in this study, evaluation of the segmentation technique was done prior to this study. Unpublished data showed an excellent surface comparison when comparing 3D models derived from 2D MRI compared to CT.

**Fig. 1:**
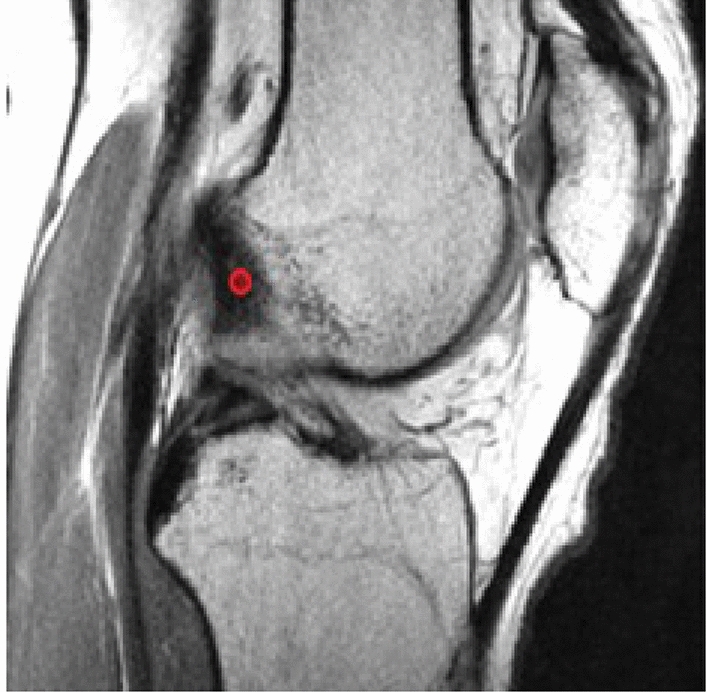
Example of sagittal view of a 3D MRI. The center of the femoral origin of the ACL was determined and marked by a red circle of 2 mm in diameter

The center of the femoral origin of the ACL was determined on the MRI images and marked by a circle of 2 mm in diameter. This point was referred to as “ACL origin,” see Fig. 1.

Previous research has shown that the identification of the femoral insertion using this method has a high intra- and interobserver reliability, even in the presence of ACL injury [29]. Intra-observer reliability for this method has been shown to be excellent with an ICC of > 0.98 and excellent interobserver reliability with an ICC of > 0.96.

**Fig. 2:**
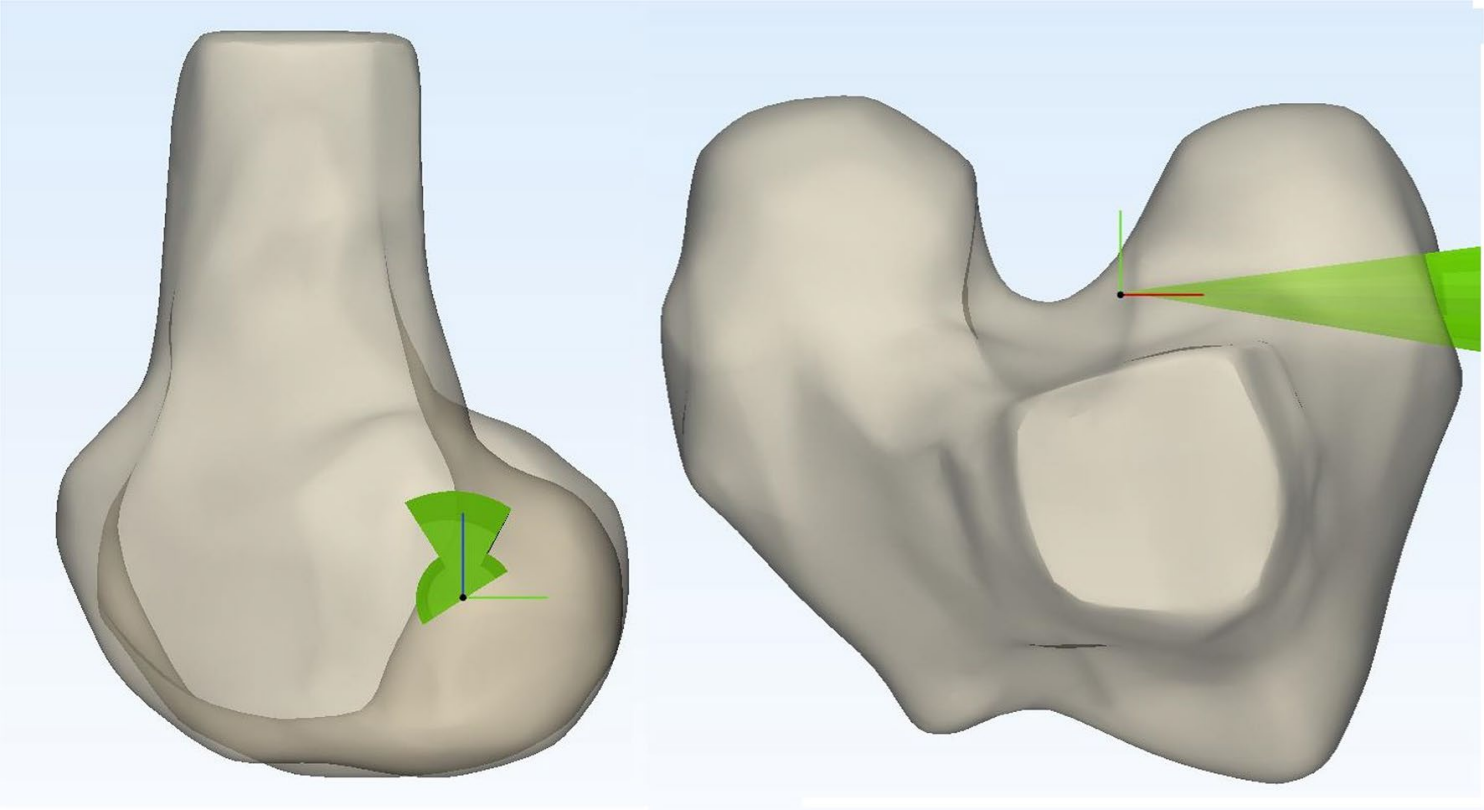
Images displaying a sagittal (left) and cranial (right) view of a 3D model of a distal femur with the cone described by Kang et al. projected in place

In order to control the drilling trajectory and ultimately the femoral tunnel position, the entry point on the lateral side of the lateral femoral condyle was selected based on the work of Kang et al. [12]. Kang recommended an optimal direction and location for the entry point of the femoral tunnel on the lateral wall of the lateral femoral condyle, taking ACL graft stress, graft bending angle and length of graft into account [12]. Based on this recommendation a cone was created, starting from the ACL origin as was determined on the medial wall of the lateral femoral condyle, projecting over the lateral aspect of the lateral condyle. This way, anatomical variation in width of the lateral femoral condyle was accounted for. See Fig. 2.

Using this technique, a point on the lateral side of the lateral femoral condyle was selected and marked by a circle of 2 mm in diameter. This point was referred to as the “entry point.” The entry point, ACL origin point, and the segmented femur were exported as Standard Tessellation Language (STL) models.

### Development of a patient specific guide

**Fig. 3:**
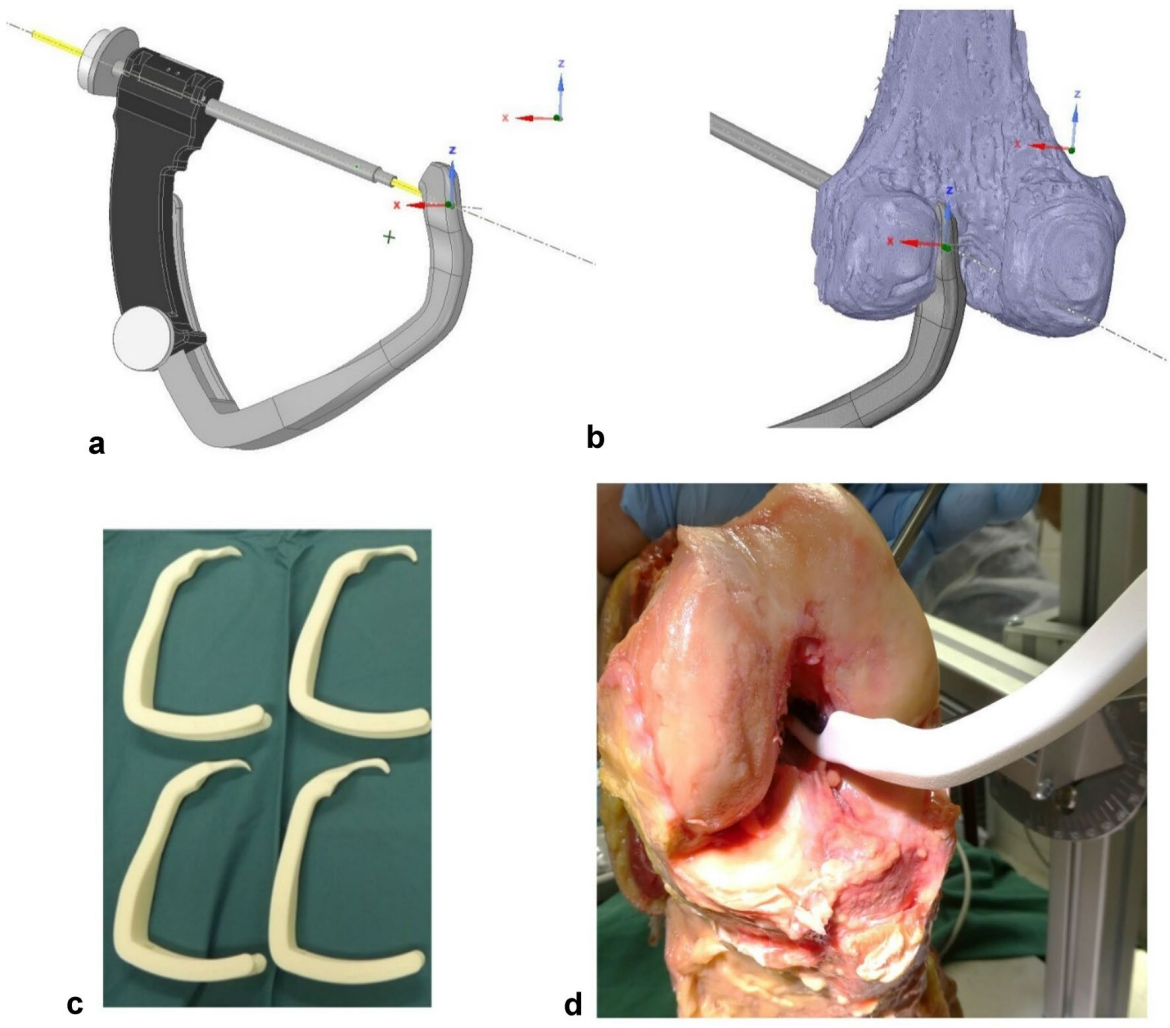
The patient specific 3D printed femoral aiming guide. **a** The drill trajectory aims for the pre-determined ACL origin **b** the aiming device fits the medial wall of the lateral femoral condyle anatomically (detailed view). **c** Inventory kit with four 3D printed PSI aiming guides. **d** Example of the 3D printed aiming guide in situ

The STL models were processed by an orthopedic engineer to create a patient specific drill guide. A negative mold of the lateral wall of the intercondylar notch was created: a box was fitted in the intercondylar notch and a Boolean operation was performed, subtracting the femoral model from the box. The drill guide was designed as an adaptation to the outside-in GraftLink^®^ technique by Arthrex using the FlipCutter^®^ (Arthrex Inc., Naples, FL, US) as described by Lubowitz [15]. The original femoral aiming guide on the Arthrex instrument was replaced by a 3D printed guide that fits the intercondylar notch, see Fig. 3.

The position of the femoral guide in combination with the 3D printed guide was designed to create a drill trajectory between the ‘entry point’ and ‘ACL origin point,’ within “Kang’s cone” see Fig. 3.

The patient specific guides were printed using a Selective Laser Sintering (SLS) printer with polyamide 12 powder (ISO 13485 certified). Polyamide 12 has an elasticity of 1650 MPa, a tensile strength of 48 MPa and was printed with a layer thickness of 0.1–0.12 mm. The material is suitable for routine steam heat sterilization by the autoclave.

### Cadaver experiment

Two male and two female cadaveric specimens were used. Average age at time of death was 88 years. Two left knees and two right knees were used. The cadavers were fixed in a custom-made leg holder. Both the femur and tibia were fixed by a clamp connected to a hinge which allowed for flexion/extension and internal/external rotation of the knee. Skin and subcutaneous tissue were dissected off. The extensor mechanism including the patella, Hoffa’s fat pad, and the anterior capsule was removed. After resection of the ACL, the patient specific hooks were introduced in the notch and were judged for its fit, see Fig. 3 (right bottom). The guides were judged upon the type of fit by two orthopedic surgeons with experience in ACL reconstruction. The type of fit was rated by each orthopedic surgeon on a 5 point Likert scale, 1 meaning a very poor fit and 5 meaning a very good fit. The orthopedic surgeons judged the type of fit independent of each other.

Next a femoral tunnel was drilled using the guide when accurate positioning based on tactile and visual feedback was confirmed.

**Fig. 4:**
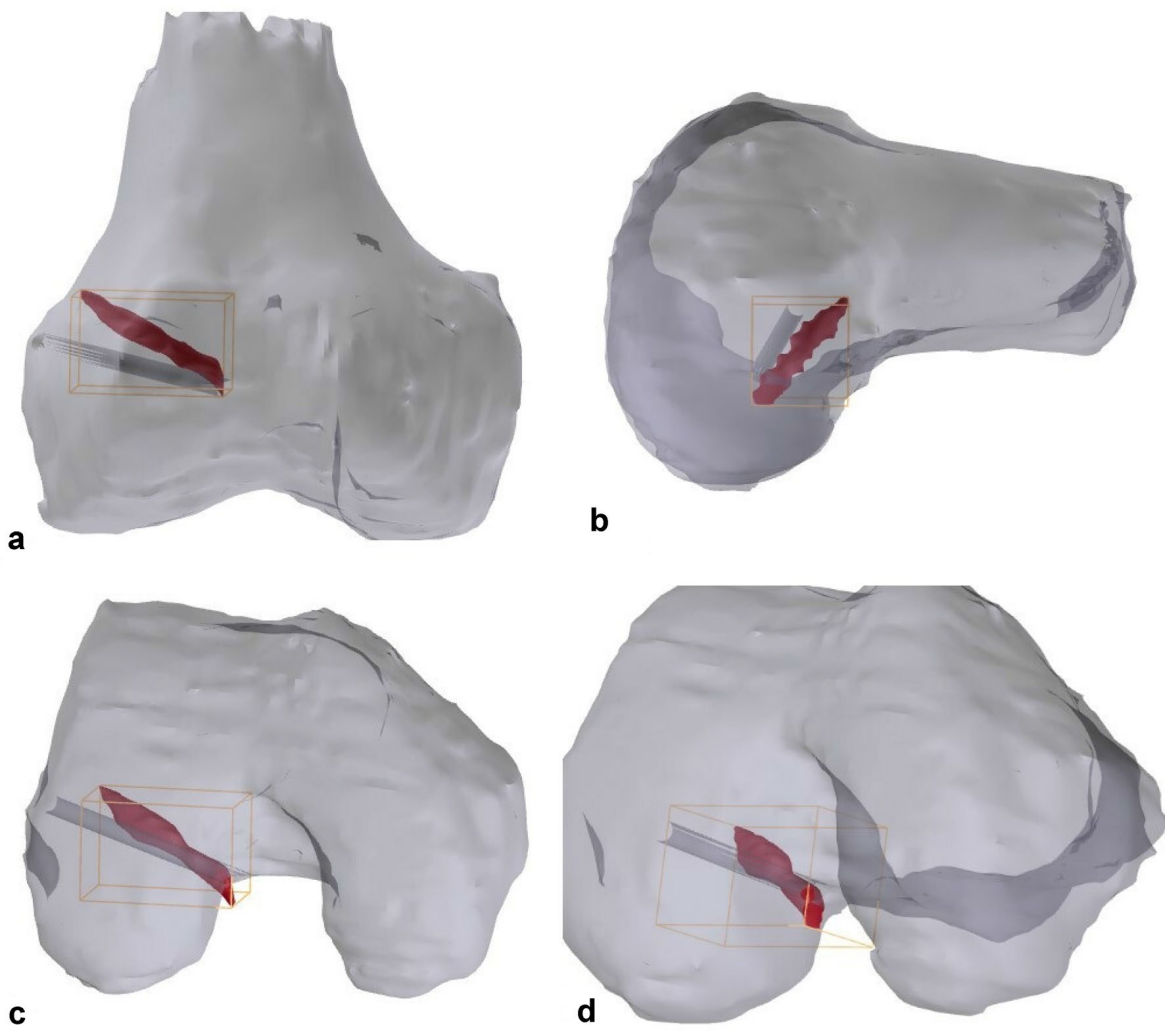
Example of comparison planned and drilled tunnels. Drilled tunnel is displayed in red. Planned tunnel in dark-gray. **a** Anterior Posterior view. **b** Sagittal view. **c** Caudo-cranial view. **d** Notch view

After the experiment, the same MRI protocol was performed as before which allowed for comparison of the actual drill trajectory with the planned drill trajectory. Both the pre- and post-procedural scans were segmented as described before. The post-procedural drill trajectories were easily identified and segmented as cylinders on all post-procedural scans (See Fig. 4). The position of these cylinders was compared to the pre-procedural planned drill trajectories. Measurements were performed in Mimics. Distances from cylinder edge to cylinder edge were recorded in mm using a digital ruler. Because of the oblique drilling trajectory, the center of the cylinder was hard to determine; therefore, we chose for edge-to-edge measurements and added the diameter of the RetroDrill (3.5 mm) to this measurement. All measurements were performed by one trained observer. The measurements were repeated by the same observer more than 2 weeks later to determine the intra-observer reliability of the measurements. The intraclass correlation coefficient (ICC 2-way random, absolute agreement) was calculated between the first and second assessment. A value less than 0.5 was considered to be indicative of poor reliability, value between 0.5 and 0.75 indicates moderate reliability, a value between 0.75 and 0.9 indicates good reliability, and a value greater than 0.90 indicates excellent reliability.

## Results

The introduced hooks provided a very good fit in the intercondylar notch as shown in Table 1. The two orthopedic surgeons reported similar results as shown in Table 1.

**Table 1:** Overview of the fitting properties of the patient specific guides as judged by the two orthopedic surgeons

Observer	Cadaver 1	Cadaver 2	Cadaver 3	Cadaver 4
Orthopedic Surgeon 1	Good	Very Good	Very Good	Very Good
Orthopedic Surgeon 2	Good	Very Good	Very Good	Very Good

Fitting properties were rated on a 5 point Likert scale (1 = very poor, 2 = poor, 3 = moderate, 4 = good, 5 = very good)

Using the 3D printed guide hooks resulted in a mean difference of 5.0 mm (SD 1.0 mm range 3.8–6.7 mm) between the planned and actual drilled trajectory. For an example, see Fig. 4. In Fig. 4, the planned drill trajectory is displayed as the dark-gray cylinder. The actual drilled tunnel is displayed in red.

The intraclass correlation coefficient for intra-observer reliability regarding the distance measurements between the planned and achieved tunnel position was calculated to be excellent: 0.956 for average measures (95% confidence interval 0.558–0.997, *p = *0.01).

## Discussion

The main finding of our study is that with our patient-specific targeting device a deviation of 5.0 mm exists compared to the planned tunnel. While the technique and development seem promising, this is outside our intended target of < 2 mm.

The accuracy of the segmentation process could be a large contributory factor to the inaccuracy of the current construct. In this study, we have segmented the MRI images semi-automatically. Even though we have observed that repeated segmentation of the same images leads to a minimal change in the total absolute difference in the model, minor impurities of the model may cause the final aiming device to fit incorrect. Nevertheless, we noticed that the fit was very good. A recent review has demonstrated the potential of automated segmentation based on deep learning [5]. As this technique develops over time, segmentation may be more accurate and less time consuming.

In addition, the construct using polyamide 12 could attribute to lower accuracy of the aiming device, since polyamide 12 contains a certain degree of flexibility. This can lead to a bending of the system. This can be solved by using more rigid materials. Titanium is available for 3D printing, but this is a costly affair. More obvious is the use of 316L stainless steel as it is used for many surgical instruments. 316L stainless steel can be machined by a robotic milling cutter to create the patient specific part for the targeting device. The use of polyamide 12, however, is a cheap option. We have not performed a cost-effectiveness analysis in this study. In this study, the total cost for a 3D virtual surgical plan and 3D printed guides were approximately 700–1000 euro per case, with approximately 100–300 euro for the 3D printed guides.

We have to conclude that so far, the total deviation has been too large, and we need further improvements to ensure that partially anatomic placement of the ACL graft will not occur.

Up to now, only one other study has been published regarding the potential of a 3D printed patient specific targeting device for the creation of the femoral tunnel. Ranking et al. have reported on a patient specific template that can be used to mark the insertion of the ACL in the notch with a chondropick [22]. Rankin et al. did not describe the accuracy of their system.

In total hip and knee arthroplasty, the use of 3D printed patient specific instruments (PSI) has shown added value in the form of high accuracy [8, 21]. However, no demonstrable improvement in patient reported outcome, and surgery time or transfusion rate has been shown when using PSI compared to standard total knee arthroplasty [13]. As exact anatomic reconstruction within a 2 mm range of the native ACL footprint already has shown to have a significant relation with graft failure, the accurateness provided using PSI in ACL reconstruction may have more noticeable effects.

The accuracy of femoral tunnel placement has been studied extensively before. An empirical optimal point for femoral tunnel position has been determined based on cadaver studies at a point at 28% on the proximal-to-distal axis and 35% on the perpendicular axis [2]. It has been shown that when surgeons rely on anatomical landmarks alone, a mean deviation of 12.5 mm occurs with respect to this empirical optimal point [10]. This emphasizes that current, widespread used surgical techniques fail to recreate the native ACL. The use of intra-operative fluoroscopy can improve accuracy, but still a mean deviation of 9.8 mm remains. Other reports show that an experienced surgeon can obtain a deviation of 4.2 mm of the femoral origin when using arthroscopy alone, which can improve to 2.7 mm when using intra-operative navigation [20]. Additive value in ACL reconstruction in terms of accuracy of femoral tunnel placement was shown using computer assisted surgery (CAS) [4, 16, 20]. The use of CAS during ACL reconstruction has been shown to lead to a deviation of planned tunnels of approximately 2 mm, in which 1 mm is attributed to the overall robotic system and 1 mm to intra-operative movement of the patient. Disadvantages of CAS include the learning curve and time consumption during surgery. With our newly developed PSI system, we strive for comparable results in terms of accuracy, while at the same time, using a simpler and more practical construct. The main difference between a CAS/Fluoroscopy-based approach and a PSI concept is that PSI strives for an individual anatomic approach rather than a one size fits all approach which leads back to an empirical determined point averaged over multiple cadaveric studies [2, 10]. It is therefore that our selected point cannot be compared to this empirical optimal point, as we never aimed for the empirical optimal point.

The shortcoming of the current surgical techniques is resembled by the high prevalence of femoral tunnel malposition. It has been recognized before that a one size fits all approach is not the way to go in ACL reconstruction [18]. Using the current available techniques that rely on the intra-operative identification of anatomical landmarks and ACL remnants, an accurate, true anatomic femoral tunnel position is not easily achieved. With the use of PSI, we aim to provide a patient specific true anatomic ACL reconstruction that does not rely on the experience of the surgeon. When both the femoral and tibial tunnel are positioned at the native origin and insertion sites, the graft can resemble the native ACL more closely.

From a practical point of view, we have chosen to aim for the center of the femoral footprint of the ACL which was regarded as the midpoint between the anteromedial (AM) and posterolateral (PL) bundle of the ACL. The advantage of the PSI design as described here is that the surgeon has ultimate control over the entire femoral tunnel position. This means that a point toward the AM bundle can be selected as well. The selected point in this experiment is not representative for clinical use as mid-bundle techniques potentially have a higher graft re-rupture rate [22]. The aim of our study was limited to determining the accuracy of the patient specific aiming guide; in other words, can we achieve a planned tunnel position. The scope of this study did not involve the amount of coverage of the ACL footprint. However, we hypothesize that recreation of native anatomy will improve outcome after ACL reconstruction. The footprint of the ACL has been shown to vary in size from 60mm2 to 130mm2, of which about half of it being reserved for each bundle [18]. An average hamstrings graft of 8 mm in diameter can cover an area of about 50mm2 (*A* = *π* *r*^2^) which increases to about 80mm2 when a 10 mm graft is harvested. More recent studies by Smigelski have shown that the ACL may in fact be more ribbon shaped [27], and ACL reconstruction techniques have been proposed to reconstruct the ACL using a ribbon shaped graft [6]. On the other hand, some authors advocate the reconstruction of the isometric, direct fibers of the ACL using the I.D.E.A.L. technique [19]. Ideally, if we strive for patient specific ACL reconstruction, the native ACL should be reconstruction in all its shape and dimensions. A recent study has shown that anthropometric data can be used to predict the graft dimensions, by which means an appropriate graft can be selected preoperatively [24]. That way true anatomic ACL reconstruction may become within reach.

Finally, we would like to emphasize that the guides in the present study have been used in a situation that replicates open surgery. This allowed for visual feedback in addition to tactile feedback in search for the optimal fit. Therefore, the results of the current study cannot be translated one-on-one to an experiment in an arthroscopic setting. The next step is to develop a guide that can be used arthroscopically. This would ask for a slimmer design which special attention to allow for easy introduction through the portal. By further improving the design, the authors hope to further improve the accuracy of the patient specific guide.

In this proof-of-concept study, the use of 3D printed patient specific instrument for anatomic ACL reconstruction has been shown feasible. An accuracy of 5 mm is demonstrated on cadavers. Currently, this is not sufficient for the instrument to be used in a human population. Further improvement in the design and materials is needed before this concept can be introduced in an in vivo setting.

## Data Availability

The data of this study are available to obtain from the corresponding author upon reasonable request.

## Abbreviations

3D: Three dimensional
ACL: Anterior cruciate ligament
AM: Anteromedial
AMP: Anteromedial portal
CAS: Computer assisted surgery
DICOM: Digital imaging and communications in medicine
ICC: Intraclass correlation coefficient
mm: Millimeter
MPa: Megapascal (pressure unit)
MRI: Magnetic resonance imaging
OI: Outside-in
PD: Proton density
PL: Posterolateral
PSI: Patient specific instruments
SD: Standard deviation
SLS: Selective laser sintering
STL: Standard tessellation language
UMCG: University Medical Center Groningen

## References

[1] Aglietti P, Buzzi R, Giron F, Simeone AJ, Zaccherotti G (1997). Arthroscopic-assisted anterior cruciate ligament reconstruction with the central third patellar tendon. A 5–8-year follow-up. Knee Surg Sports Traumatol Arthrosc.

[2] Bird JH, Carmont MR, Dhillon M (2011). Validation of a new technique to determine midbundle femoral tunnel position in anterior cruciate ligament reconstruction using 3-dimensional computed tomography analysis. Arthroscopy.

[3] Brandsson S, Karlsson J, Sward L, Kartus J, Eriksson BI, Karrholm J (2002). Kinematics and laxity of the knee joint after anterior cruciate ligament reconstruction: pre- and postoperative radiostereometric studies. Am J Sports Med.

[4] Burkart A, Debski RE, McMahon PJ (2001). Precision of ACL tunnel placement using traditional and robotic techniques. Comput Aided Surg.

[5] Ebrahimkhani S, Jaward MH, Cicuttini FM, Dharmaratne A, Wang Y, de Herrera AGS (2020). A review on segmentation of knee articular cartilage: from conventional methods towards deep learning. Artif Intell Med.

[6] Fink C, Smigielski R, Siebold R, Abermann E, Herbort M (2020). Anterior cruciate ligament reconstruction using a ribbon-like graft with a C-shaped tibial bone tunnel. Arthrosc Tech.

[7] Wright RW, Huston LJ, Group M (2010). Descriptive epidemiology of the multicenter ACL revision study (MARS) cohort. Am J Sports Med.

[8] Hananouchi T, Saito M, Koyama T, Sugano N, Yoshikawa H (2010). Tailor-made surgical guide reduces incidence of outliers of cup placement. Clin Orthop Relat Res.

[9] Hayback G, Raas C, Rosenberger R (2022). Failure rates of common grafts used in ACL reconstructions: a systematic review of studies published in the last decade. Arch Orthop Trauma Surg.

[10] Inderhaug E, Larsen A, Waaler PA, Strand T, Harlem T, Solheim E (2017). The effect of intraoperative fluoroscopy on the accuracy of femoral tunnel placement in single-bundle anatomic ACL reconstruction. Knee Surg Sports Traumatol Arthrosc.

[11] Jaecker V, Zapf T, Naendrup JH, Kanakamedala AC, Pfeiffer T, Shafizadeh S (2018). Differences between traumatic and non-traumatic causes of ACL revision surgery. Arch Orthop Trauma Surg.

[12] Kang K, Bae TS (2017). Effect of femoral tunnel positions on graft stress in outside-in ACL reconstruction surgery during continuous knee motion: a simulation study. Int J Med Robot Comput Assist Surg.

[13] Kizaki K, Shanmugaraj A, Yamashita F (2019). Total knee arthroplasty using patient-specific instrumentation for osteoarthritis of the knee: a meta-analysis. BMC Musculoskelet Disorders.

[14] Logan M, Dunstan E, Robinson J, Williams A, Gedroyc W, Freeman M (2004). Tibiofemoral kinematics of the anterior cruciate ligament (ACL)-deficient weightbearing, living knee employing vertical access open "interventional" multiple resonance imaging. Am J Sports Med.

[15] Lubowitz JH, Ahmad CS, Anderson K (2011). All-inside anterior cruciate ligament graft-link technique: second-generation, no-incision anterior cruciate ligament reconstruction. Arthroscopy.

[16] Luites JW, Wymenga AB, Blankevoort L, Eygendaal D, Verdonschot N (2014). Accuracy of a computer-assisted planning and placement system for anatomical femoral tunnel positioning in anterior cruciate ligament reconstruction. Int J Med Robot Comput Assist Surg.

[17] Meuffels DE, Poldervaart MT, Diercks RL (2012). Guideline on anterior cruciate ligament injury. Acta Orthop.

[18] Musahl V, Nazzal EM, Lucidi GA (2022). Current trends in the anterior cruciate ligament part 1: biology and biomechanics. Knee Surg Sports Traumatol Arthrosc.

[19] Pearle AD, McAllister D, Howell SM (2015). Rationale for strategic graft placement in anterior cruciate ligament reconstruction: IDEAL femoral tunnel position. Am J Orthop (Belle Mead NJ).

[20] Picard F, DiGioia AM, Moody J (2001). Accuracy in tunnel placement for ACL reconstruction. Comparison of traditional arthroscopic and computer-assisted navigation techniques. Comput Aided Surg.

[21] Popescu D, Laptoiu D (2016). Rapid prototyping for patient-specific surgical orthopaedics guides: A systematic literature review. Proc Inst Mech Eng Part H.

[22] Rankin I, Rehman H, Frame M (2018). 3D-printed patient-specific ACL femoral tunnel guide from MRI. The Open Orthop J.

[23] Ristanis S, Giakas G, Papageorgiou CD, Moraiti T, Stergiou N, Georgoulis AD (2003). The effects of anterior cruciate ligament reconstruction on tibial rotation during pivoting after descending stairs. Knee Surg Sports Traumatol Arthrosc.

[24] Sadoghi P, Roggla V, Beiglbock H (2023). Prediction of individual graft for anterior cruciate ligament reconstruction using anthropometric data. Arch Orthop Trauma Surg.

[25] Scheffler SU, Maschewski K, Becker R, Asbach P (2018). In-vivo three-dimensional MR imaging of the intact anterior cruciate ligament shows a variable insertion pattern of the femoral and tibial footprints. Knee Surg Sports Traumatol Arthrosc.

[26] Sim JA, Na YG, Choi JW, Lee BH (2022). Early medial reconstruction combined with severely injured medial collateral ligaments can decrease residual medial laxity in anterior cruciate ligament reconstruction. Arch Orthop Trauma Surg.

[27] Smigielski R, Zdanowicz U, Drwiega M, Ciszek B, Williams A (2016). The anatomy of the anterior cruciate ligament and its relevance to the technique of reconstruction. Bone Jt J.

[28] van Eck CF, Widhalm H, Murawski C, Fu FH (2015). Individualized anatomic anterior cruciate ligament reconstruction. Phys Sportsmed.

[29] Zee MJM, Sulaihem RA, Diercks RL, van den Akker-Scheek I (2021). Intra-and interobserver reliability of determining the femoral footprint of the torn anterior cruciate ligament on MRI scans. BMC Musculoskelet Disorders.

